# Supplementary material for: Cross-cultural adaptation and psychometric properties of the Italian version of the Body Perception Questionnaire
Source: PLoS One. 2021 May 27;16(5):e0251838. doi: 10.1371/journal.pone.0251838 (PMC8158925; doi:10.1371/journal.pone.0251838)
Supplement: S1 File — (DOCX) [file pone.0251838.s001.docx]

**S1 Table. Counts of pathologies and medication users**

|  | Count | Percent (%) |
| --- | --- | --- |
| Pathologies |  |  |
| Cancer | 2 | 1.49 |
| Cardiovascular | 25 | 4.64 |
| Cranio-Facial | 13 | 2.41 |
| Dermatological | 18 | 3.34 |
| Gastro-Intestinal | 24 | 4.45 |
| Haematological | 3 | 0.56 |
| Hormonal | 24 | 4.45 |
| Neurologic | 11 | 2.04 |
| Others | 28 | 5.19 |
| Respiratory | 17 | 3.15 |
| Rheumatic | 8 | 1.49 |
| Urogenital | 15 | 2.78 |
| Medications |  |  |
| Antibiotics | 9 | 1.67 |
| Cardiovascular | 24 | 4.45 |
| Immunosuppressor | 1 | 0.19 |
| Metabolics | 29 | 5.38 |
| NSAIDs or Corticosteroids | 16 | 2.97 |
| Others | 51 | 9.46 |
| Psychoactive | 8 | 1.48 |
| Respiratory | 12 | 2.23 |

**S2 Table. Awareness Linear Model**

| Characteristic | β | 95% C.I. | P-value |
| --- | --- | --- | --- |
|  |  |  |  |
| Age | -0.06 | -0.09 to -0.02 | 0.002 |
| Gender (M) | -1.28 | -2.33 to -0.23 | 0.02 |
| Medications (Yes) | 1.01 | -0.16 to 2.19 | 0.09 |
| Physical Activity (≥2 times/week) | -0.2 | -1.27 to 0.86 | 0.7 |

**S3 Table. ANSR Supradiaphragmatic Linear Model**

| Characteristic | β | 95% C.I. | P-value |
| --- | --- | --- | --- |
|  |  |  |  |
| Age | -0.03 | -0.06 to -0.004 | 0.02 |
| Gender (M) | -1.05 | -1.81 to -0.29 | 0.007 |
| Physical Activity (≥2 times/week) | -1.66 | -2.43 to -0.89 | <0.001 |
| Medications (Yes) | 0.97 | 0.12 to 1.82 | 0.03 |

**S4 Table. ANSR Subdiaphragmatic Linear Model**

| Characteristic | β | 95% C.I. | P-value |
| --- | --- | --- | --- |
|  |  |  |  |
| Age | -0.02 | -0.03 to -0.005 | 0.004 |
| Gender (M) | -0.60 | -0.93 to -0.26 | 0.0005 |
| Physical Activity (≥3 times/week) | -0.44 | -0.79 to -0.1 | 0.01 |
| Medications | 0.29 | -0.08 to 0.67 | 0.16 |

**S1 Text. Exploratory Factor Analysis description.**

Exploratory Factor Analysis (EFA) items were scored using a 5 point likert scale (1 = never, 2 = rarely, 3 = sometimes, 4= often, 5 = always) in order to maximize sensitivity for individual differences.

EFA was conducted for the full-scoring system, in order to provide alternative results, which may be more sensitive for assessing individual differences. The EFA analysis were conducted on two polychoric correlation matrices, one for Awareness model (26x26) and one for ANSR model (20x20), using the fa function of “psych” R package [1]. Model fit, factor loading simple structure, theoretical predictions, and scree plots guided exploratory factor retention [2]. The oblique rotation (“oblimin” in R coding) was used. Root mean squared error of approximation (RMSEA), the Tucker‐Lewis Index (TLI), and the Comparative Fit Index (CFI) was adopted to evaluate the Goodness of fit to the data [3–6].We interpreted good fit to be evidenced by an RMSEA value near .06 or lower as well as CFI and TLI values near .95 or greater, as recommended by Hu and Bentler [7]. The number of the factors in each model was selected using parallel analysis of the scree plot, using the observed eigenvalues that substantially deviated from resampled and simulated eigenvalues [8].

EFA findings were retrieved by parallel analysis of the scree plots suggesting that body awareness could be described by one factor and autonomic reactivity by two factors (Fig. 1, 2). Notably, the goodness of fit indices for these solutions did not reach good fit BPQ-I (RMSEA=0.11, CFI=0.79 , TLI=0.77 for Awareness model, and RMSEA=0.08, CFI=0.85 , TLI=0.82 for ANSR model). The Chi-squared and the degrees of freedom were returned for each model ( χ2= 1351.14 ,df=299 for Awareness model; χ2=830.94, df=274 for ANSR model). While additional factors improved fit, their inclusion resulted in solutions with evidence of over-factoring where some items did not have any substantial loadings. Thus, the 1-factor body awareness and 2-factor autonomic reactivity solution were deemed to be optimal.

**S1 Figure. Exploratory** **parallel analysis for body awareness.**

**
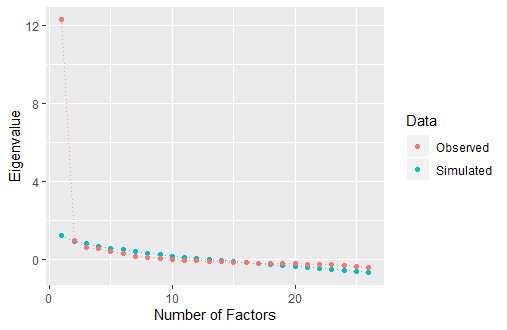
**

**S2 Figure. Exploratory parallel analysis for** **the autonomic nervous system reactivity domain.**

**
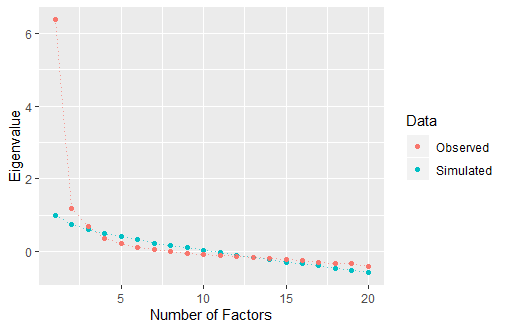
**

**S5 Table. Exploratory (EFA) and confirmatory (CFA) factor analysis standardized factor loadings**

| **BPQ Items** |  | **EFA** |  | **CFA** |
| --- | --- | --- | --- | --- |
|  |  |  |  |  |
| **Awareness** |  |  |  |  |
|  |  |  |  |  |
| “Swallowing frequently” |  | 0.5 |  | 0.47 |
| “An urge to cough to clear my throat” |  | 0.58 |  | 0.47 |
| “My mouth being dry” |  | 0.67 |  | 0.54 |
| “How fast I am breathing” |  | 0.68 |  | 0.55 |
| “Watering or tearing of my eyes” |  | 0.69 |  | 0.54 |
| “Noises associated with my digestion” |  | 0.71 |  | 0.51 |
| “A swelling of my body or parts of my body” |  | 0.67 |  | 0.47 |
| “An urge to defecate” |  | 0.74 |  | 0.51 |
| “Muscle tension in my arms and legs” |  | 0.68 |  | 0.53 |
| “A bloated feeling because of water retention” |  | 0.49 |  | 0.43 |
| “Muscle tension in my face” |  | 0.67 |  | 0.58 |
| “Goose bumps” |  | 0.79 |  | 0.56 |
| “Stomach and gut pains” |  | 0.73 |  | 0.48 |
| “Stomach distension or bloatedness” |  | 0.65 |  | 0.52 |
| “Palms sweating” |  | 0.72 |  | 0.59 |
| “Sweat on my forehead” |  | 0.77 |  | 0.63 |
| “Tremor in my lips” |  | 0.83 |  | 0.66 |
| “Sweat in my armpits” |  | 0.73 |  | 0.52 |
| “The temperature of my face (especially my ears)” |  | 0.7 |  | 0.56 |
| “Grinding my teeth” |  | 0.59 |  | 0.49 |
| “General jitteriness” |  | 0.7 |  | 0.48 |
| “The hair on the back of my neck standing up” |  | 0.66 |  | 0.53 |
| “Difficulty in focusing” |  | 0.62 |  | 0.46 |
| “An urge to swallow” |  | 0.71 |  | 0.61 |
| “How hard my heart is beating” |  | 0.75 |  | 0.56 |
| “Feeling constipated” |  | 0.7 |  | 0.53 |
|  |  |  |  |  |
|  |  |  |  |  |
| **ANSR** |  | **Supra; Sub** |  | **Supra; Sub** |
|  |  |  |  |  |
| “I have difficulty coordinating breathing and eating” |  | 0.76; -0.07 |  | 0.61; 0 |
| “When I am eating, I have difficulty talking” |  | 0.51; 0.01 |  | 0.53; 0 |
| “My heart often beats irregularly” |  | 0.45; 0.18 |  | 0.52; 0 |
| “When I eat, food feels dry and sticks to my mouth and throat” |  | 0.49; 0.14 |  | 0.58; 0 |
| “I feel shortness of breath” |  | 0.58; 0.09 |  | 0.57; 0 |
| “I have difficulty coordinating breathing with talking” |  | 0.70; -0.08 |  | 0.60; 0 |
|  |  |  |  |  |
| “When I eat, I have difficulty coordinating swallowing, chewing, and/or sucking with breathing” |  | 0.80; -0.16 |  | 0.61; 0 |
| “I have a persistent cough that interferes with my talking and eating” |  | 0.52; 0.13 |  | 0.57; 0 |
| “I gag from the saliva in my mouth” |  | 0.51; 0.15 |  | 0.62; 0 |
| “I have chest pains” |  | 0.39; 0.24 |  | 0.55; 0 |
| “I gag when I eat” |  | 0.48; 0.20 |  | 0.58; 0 |
| “When I talk, I often feel I should cough or swallow the saliva in my mouth” |  | 0.57; 0.09 |  | 0.60; 0 |
| “When I breathe, I feel like I cannot get enough oxygen” |  | 0.59; 0.08 |  | 0.61; 0 |
| “I have difficulty controlling my eyes” |  | 0.38; 0.29 |  | 0.54; 0 |
| “I feel like vomiting” |  | 0.30; 0.46 |  | 0.39; 0.29 |
|  |  |  |  |  |
| ASNR Subdiaphragmatic |  |  |  |  |
|  |  |  |  |  |
| “I feel like vomiting” |  |  |  |  |
| “I have 'sour' stomach” |  | -0.03; 0.69 |  | 0; 0.61 |
| “I am constipated” |  | 0.07; 0.49 |  | 0; 0.62 |
| “I have indigestion” |  | 0.03; 0.74 |  | 0; 0.70 |
| “After eating I have digestive problems” |  | -0.05; 0.72 |  | 0; 0.66 |
| “I have diarrhea” |  | 0.08; 0.45 |  | 0; 0.55 |

References

1. Revelle W. psych: Procedures for Psychological, Psychometric, and Personality Research. 2020. Available: https://CRAN.R-project.org/package=psych

2. Cattell RB. The Scree Test For The Number Of Factors. Multivar Behav Res. 1966;1: 245–276. doi:10.1207/s15327906mbr0102_10

3. Steiger JH. Structural Model Evaluation and Modification: An Interval Estimation Approach. Multivar Behav Res. 1990;25: 173–180. doi:10.1207/s15327906mbr2502_4

4. Steiger JH. Notes on the Steiger–Lind (1980) Handout. Struct Equ Model Multidiscip J. 2016;23: 777–781. doi:10.1080/10705511.2016.1217487

5. Tucker LR, Lewis C. A reliability coefficient for maximum likelihood factor analysis. Psychometrika. 1973;38: 1–10. doi:10.1007/BF02291170

6. Bentler PM. Comparative fit indexes in structural models. Psychol Bull. 1990;107: 238–246. doi:10.1037/0033-2909.107.2.238

7. Hu L, Bentler PM. Cutoff criteria for fit indexes in covariance structure analysis: Conventional criteria versus new alternatives. Struct Equ Model Multidiscip J. 1999;6: 1–55. doi:10.1080/10705519909540118

8. Fabrigar LR, Wegener DT, MacCallum RC, Strahan EJ. Evaluating the use of exploratory factor analysis in psychological research. Psychol Methods. 1999;4: 272–299. doi:10.1037/1082-989X.4.3.272
