# Supplementary material for: Cross-cultural adaptation and psychometric properties of the Italian version of the Body Perception Questionnaire
Source: PLoS One. 2021 May 27;16(5):e0251838. doi: 10.1371/journal.pone.0251838 (PMC8158925; doi:10.1371/journal.pone.0251838)
Supplement: Attachment — Submitted filename: Review_PONE-D-19-35072.pdf [file pone.0251838.s002.pdf]

General Comments

In this manuscript, the authors describe the cross-cultural adaptation and psychometric analysis of the Italian version of the Body Perception Questionnaire (BPQ). They also provide a subsequent analysis exploring the association between the BPQ and demographic/clinical variables. This work is extremely relevant and important for researchers working with body awareness, interoception, autonomic reactivity, embodied cognition, social cognition, among several other fields. There is a clear need to validate and explore the psychometric proprieties of body awareness scales for different languages, especially because there is a lot of debate in the literature about what these questionnaires are truly measuring. For instance, to my knowledge, there is only one psychometric study about the BPQ. One of the major strengths of this work is an enormously thorough cross-cultural adaption methodology. Properly translating a questionnaire about perceiving internal bodily sensations is an extremely difficult task and the authors were extremely careful when addressing this challenge. The authors were also able to achieve a quite large and adequate sample size for psychometric analysis. However, after reading the manuscript, I felt that this version is still quite preliminary and needs a lot of work. It was hard to follow the manuscript as each section was not very well structured, there were too many inconsistencies in terminology and several references misplaced. Written expression was reasonable, although it can still be improved. Furthermore, there are also several issues regarding statistical analysis, theoretical framework about body awareness, and study limitations that I would like to debate in the authors. My review is quite long and throughout because I believe this work is extremely valuable and should be improved to high-quality standards so that it can be useful for researchers in this field.

1. Define study goals more clearly

1.1. After reading the manuscript it seemed to me that this study had three main goals:

- Cross-cultural adaptation of the BPQ Short-Form for Italian.
- Examine the psychometric properties of the Italian BPQ Short-Form
- Examine the association between BPQ subscales and sociodemographic/clinical variables (e.g. age, BMI, smoking, diagnosis, medication intake, etc).

These goals should be stated at the end of the Introduction section and clearly noticed by the reader across the manuscript.

1.2. Clinimetric vs Psychometric: I know this is just a terminology issue, but I was surprised when the authors described their study as clinimetric. The terms are widely overlapping (De Vet *et al.* 2003 - 10.1016/j.jclinepi.2003.08.010) and some argue that clinimetrics is not a unique approach,

but is rather a subset of psychometrics (Streiner, 2003 - 10.1016/j.jclinepi.2003.08.011). I believe that this term is not very frequently used nowadays (a quick search on PubMed records in the last 5 years confirmed this). I would suggest that the authors use the term psychometric study as it is much more widely used.

2. Improve the Introduction section: Although the introduction provides valuable information about body awareness and how this concept is linked to other phenoms (e.g. mental illness, management of chronic diseases), I still felt that this section was a little bit confusing and that some information was lacking.

2.1. Although I know that the terminology in this field is quite heterogenous, it would be important for me to clearly understand whether the authors consider body awareness and body perception similar constructs. The authors kind of state that at line 61 (page 3), but if the authors truly consider them as similar this should be explicitly stated at the start of the Introduction section. I actually believe it would be easier simply to talk about body awareness for consistency purposes, especially because it is one of the BPQ subscales.

2.2. The introduction was mainly focused on defining and debating body awareness. This allows the reader to understand the body awareness domain, but not the autonomic nervous system reactivity subscale. Actually, I felt that this subscale was not evidently explained across the whole manuscript. The authors should clearly define both body awareness and automatic reactivity. I do not think this is an issue as the BPQ subscales are proposed to assess different domains and not a single unitary construct.

2.3. The authors introduced the concept of interoception and its association with body awareness and rightfully so in my opinion, as the BPQ is widely used as a measure for interoception in behavioral sciences. However, I believe the authors should further explore the most recently debated models about interoception when addressing this topic. This might seem unnecessary or not valuable for the main goals of this work, but in my opinion, this discussion is key when addressing the validity of this scale (especially for the body awareness subscale) and could help to interpret the results obtained by the authors. (more information below). If the authors feel that this information is not required in the Introduction, this should at least be debated in the Discussion section. Please check these three overviews about interoception measurement (the main ones that I know of in the last 5 years).

- Garfinkel *et al*. (2015) - 10.1016/j.biopsycho.2014.11.004

Argues that interoception encompasses three distinct dimensions (accuracy, sensibility, and awareness). According to these authors, the BPQ Body Awareness subscale measures interoceptive sensibility.

- Khalsa *et al*. (2017) – 10.1016/j.bpsc.2017.12.004

Proposed that interoceptive awareness encompasses a complex taxonomy (attention, detection, magnitude, discrimination, accuracy, sensibility, insight, self-report). This work also provides a lot of information about the link between interoception and mental health which I think is relevant for this manuscript.

- Murphy *et al.* (2019) – 10.3758/s13423-019-01632-7

Proposes a 2 x 2 factorial structure of interoception (factor 1: what is measured – accuracy vs. attention; factor 2: how is it measures – beliefs vs. performance). According to the authors, BPQ measures beliefs about interoceptive attention.

2.4. The authors decided to differentiate body awareness from somatosensory amplification. Although this is interesting from a conceptual standpoint, I am not sure if it really had a lot of valuable information as the authors do not address this concept in any other section.

2.5. I think that the structure of the introduction should be rethought for the purposes of clarity. The section is not very fluid. Reorganizing the several pieces while using some subheadings will likely make it substantially better and easier to follow. Thus, a possible structure would be (although there are other alternatives that may better suit the authors):

- Define body awareness (including its link to interoception) and autonomic reactivity;
- Describe the physiological circuits of body awareness and autonomic reactivity (including the polyvagal theory);
- Describe the importance of body awareness and autonomic reactivity for clinical disorders (e.g. mental health, other chronic disorders, etc) and for patient subjective reports;
- Highlight the lack of studies addressing the psychometric proprieties of measures related to body awareness and automatic reactivity. Introduce the BPQ.
- Study Goals

3. Organization of Methods Section

I do not think the Methods section is very well organized. The authors seem to go back and forth between the different stages of the study. Maybe try to follow something like:

- Participants (only describe participant recruitment, sample size, and participant characteristics)
- Measures
    - BPQ: Start by describing the BPQ more clearly. This is not done in any section of the manuscript. For instance, the authors never mention that this questionnaire is completed through a 5-level Likert scale that rates frequency.
    - Describe demographic and clinical data collected. End this section by provided information about the questionnaire application online
- Cross-Cultural Adaptation
- Statistical Procedures (provide an initial overview of statistics -info in lines 173-178 - before describing each specific step)
    - Data Preparation
    - Factor Analysis and Measurement Invariance Analysis
    - Internal Consistency
    - Association between BPQ and demographic/clinical variables
    - Floor and ceiling effects

4. Sample Characteristics

4.1. The authors state that they are assessing the BPQ validity in a sample of healthy subjects. However, I could easily argue that this not very accurate. First, all participants were contacted during clinical appointments with osteopaths, most of them probably due to some health-related condition. Secondly, around 30% of your sample reported some sort of disease and almost 25% reported medication intake. Although there is no specific information about the number of subjects per type of disease (this should also be added, at least in the supplemental materials), I think that considering this sample “healthy” is a little bit farfetched. I do not have any clear suggestions for the correct terminology that the authors should use, but clinical-sample or sample of health care services users would be more accurate in my opinion.

4.2. The recruited sample is quite well-educated (40% with a university degree). Furthermore, about 30% of the participants had no income. These subjects with no income where mostly university students or just unemployed/retired? This should also be clear in my opinion as it can also strengthen the fact that this sample had above-average education levels. High education levels may be a possible source of bias that should be briefly introduced when describing study limitations.

5. Limitations of Online Surveys Should Be Addressed

5.1. The authors state the questionnaire was administered online, but there is not any more information about this. The authors should provide information on factors that are known to influence data quality in online surveys such as: average time of completion (probably not an issue in this study but should be reported); incentive for study completion (I guess there was not but this should be stated); missing values (most online studies do not have this issue but this should be stated as well); other factors the authors consider can influence data quality.

5.2. The authors did not describe any strategy to ensure data quality and exclude inadequate respondents. This may seem a minor issue, but there is evidence suggesting that careless respondents can range between 3 to 60% in self-report studies. Nowadays there are several methods to address this issue (for more information check Curran 2016 - 10.1016/j.jesp.2015.07.006 - and Niessen *et al.* 2016 - 10.1016/j.jrp.2016.04.010), but I recommend that the authors at least exclude subjects based on response time. Niessen *et al.* (2016) reported that response time was one of the most effective methods to detect careless responding. When subjects take more time to complete the questionnaire, data quality is better and there is less probability of careless responding. The authors should establish a cut off criteria to exclude subjects based on response time. There are also several suggestions about which criteria to use (e.g. three-second-per-item rule, exclude subjects 3 standard deviations below the average response time, etc) and the authors should select the criteria they feel it is more accurate (and justify it with previous studies). If the authors have not recorded response times, please look at other statistical procedures (e.g. multivariate outlier analysis). The authors may also check if there were answers submitted from duplicate IP addresses, although in this case, this seems to be highly unlikely.

5.3. Finally, the limitations of online surveys should be addressed in the Discussion section. Although participants were directly contacted, I am assuming that only subjects with internet access could participate (unless the authors had any solution to address this). This introduces a slight bias that should be referred (for instance, older participants are more likely to have no internet access).

6. Issues with Psychometric Analysis

Although the psychometric analysis is quite interesting, I would like to clarify several issues with the authors:

6.1. Construct Validity: The authors argue that they will achieve construct validity through hypothesis testing and propose some hypotheses in the Methods sections without providing previous evidence for them. This evidence should be quite strong and from several sources ideally. I would like the authors to argue whether there is clear evidence about the relationship between body awareness and automatic reactivity with age, but more importantly to the other proposed variables such as smoking, physical activity, BMI, and educational level. Furthermore, why did the authors did not include gender in this hypothesis testing as they address it on the Results and the Discussion section? Regardless, I do not believe that this strategy is sufficient for construct validity, especially pertaining to body awareness as there are so many authors arguing how interoception encompasses several measurement dimensions (as described above). This is the main issue of this work in my opinion. For me, the only alternative to accomplish this would be to examine convergent and/or discriminant validity. For instance, Cabrera et al. (2017) used the Stress Reactivity Index and SomatoSensory Amplification Scale for convergent validity. As several authors also consider that the body awareness subscale assesses interoceptive sensibility or interoceptive attention, another alternative would be using interoception questionnaires to test for convergent/discriminant validity. Alexithymia could also be measured together with interoception as a recent meta-analysis from Trevisan et al. (2019) found that the BPQ was positively associated with alexithymia, although other measures of interoception were negatively associated (10.1037/abn0000454). Finally, depression and anxiety scales could also be an interesting alternative as they have been differentially associated with interoceptive scales, including the BPQ (e.g. Murphy et al. 2019 - 10.1177/1747021819879826). There should also be several other interesting alternatives for convergent or discriminant validity regarding the automatic reactivity subscale. Basically, if the authors want to dramatically improve their work and provide clear evidence that the Italian BPQ is truly measuring body awareness and automatic reactivity is to complete another study with a smaller sample size (I would estimate about 100 although a power analysis would be needed). If this is not possible for the authors, then they should acknowledge that this work is extremely limited to provide evidence for construct validity-and provide recommendations for future studies to test this.

6.2. Although it is not my core expertise, I have some questions to be clarified/improved by the authors regarding the Confirmatory Factor Analysis (CFA) and Measurement invariance analysis

(MIA). First, the description of the CFA procedures in the Methods section seems too broad. Further details should be given to the reader (e.g. type of matrix used, estimation procedure, etc), at least in Supplemental material. Second, the indicators chosen for Table 2 regarding the CFA are not very usual. If I understood correctly, the estimates are the unstandardized factor loadings, with their corresponding standard errors. Why did the authors select not to report the standardized loadings (more common) as it was done for instance by Cabrera et al. (2017)? Is it actually necessary to report “P(>|z|)| as the values are similar for each item? For more information about reporting a CFA I advise the authors to consult the work from Jackson et al. 2009 - 10.1037/a0014694. Third, I wanted to know why the authors selected to report the Chi-Square Statistics for the MIA but not for the CFA. Conversely, why did the authors report the TLI for the CFA but not for the MIA. This should be explained to the reader.

6.3. In lines 181-183 the authors state that they dichotomized ratings as it was done on previous psychometric analysis of the BPQ (I believe the authors are refereeing to the paper from Cabrera et al. 2017, although the reference numbers seem incorrect). This is a valid argument, however, in the work from Cabrera *et al.* 2017 the authors actually decided to dichotomize the items as the exploratory factor analysis using full item distributions resulted in a large number of factors and loadings with complex factors. Did the authors perform any sort of factor analysis for the full-item distribution in this proposed Italian version? If so, this should be addressed somewhere (even if it is in the supplements). If not, I believe it will be important for the authors to complete this analysis. If the questionnaire was applied with a 5-point Likert scale, I believe that this should not be simply left out because it can provide valuable information for future studies.

6.4. Item “I feel like vomiting”: The authors only address the fact that this item was included on both reactivity subdomains in the Descriptive Statistics heading. This should be already explained in the Methods sections. Also, I think the authors should justify why did they select to keep the item on both subdomains In the work of Cabrera *et al.* (2017) the supra- and subdiaphragmatic reactivity loadings were a problem in the exploratory factor analysis of the Spanish sample. However, they also described that in the CFA factor loading of the American sample the item was associated more strongly with subdiaphragmatic reactivity. Thus, I think the authors should clearly explain why this item was retained on both subdomains in the Italian version.

6.5. The authors did not report the correlations between the supra- and sub-diaphragmatic reactivity scores with the body awareness score. This should be added.

6.6. In the internal consistency subheading, the authors should also report the confidence intervals for McDonald's omega.

7. Testing the association between BPQ and demographic/clinical variables.

7.1. In the methods section (lines 217-220, page 10) the authors should state that subscale scores were calculated based on dichotomized items. Here the authors should also briefly describe which regression models were tested (how many, dependent, and independent variables).

7.2. I would also like to know why the authors decided to use both Pearson and Kendall correlations, which are parametric and non-parametric, respectively. Was that the reason? If so, did the authors did any kind of assumption testing before deciding to select both?

7.3. In the results section, the authors describe the association between the BPQ subscales and each demographic/clinical variable. This is quite confusing for the reader. I do not understand if they prepared a regression model for each predictor or if they had a complex model with all the predictors. For the purpose of clarity, I think the authors should organize this section by first describing the significant correlations found (based on Table 6) and only them report the results of regression models (assuming the authors had one model with all predictors).

7.4. Table 6 should be presented as a correlation matrix. The way it is presented now is quite confusing. Statistically significant correlations should be highlighted in some way.

7.5. The authors reported correlations for (supposedly) categorical variables such as Smoking and Physical Activity. Computing correlations is not very advisable here in my opinion. The more adequate statistical approach would be to compare Smoker vs. Non-Smokers or Physical Active vs. Inactive using independent samples t-tests.

7.6. I think that Figure 1 is not necessary as this correlation could be merely reported in Table 6 and in the text.

7.7. I have several issues with the linear models sent in the supplementary material, which are actually described in the manuscript. First, I do not understand why the authors computed a regression model with the BPQ total score. The authors do not talk about a BPQ total score across the whole manuscript and I do not think the BPQ was designed to have a total score. At line 300,

page 15 the authors also address this model. This analysis should not be reported in my opinion. Secondly, the authors presented linear models with more than 20 predictors. I do not think that the sample size is adequate for this analysis. Third, the authors do not report how many subjects have each pathology. For instance, if you include psychiatric disorders as a predictor, there are only 4 subjects with a diagnosis. This cannot provide valuable insights in a regression model. This issue is probably true for other diagnoses, but the authors must clarify how many subjects are in each type of pathology. If the authors are interested in exploring the association between BPQ and diagnosis, maybe it would be easier to perform independent group t-tests for each diagnostic group (only for groups with significant sample size) and report mean and standard deviation for each group. You can even control the p-value for multiple comparisons. Mann–Whitney U tests may also be used if the sample size is reduced and/or normality assumption is not met

7.8. At line 311, Page 16 the authors describe an ANOVA analysis that was never mentioned in the Methods sections.

8. Enhance the Discussion section

8.1. The first paragraph (lines 321-323, page 16) should reinforce the 3 main goals that I argued at the beginning of my review. As it is now written it seems to empathize only internal consistency which is only a part of the authors work.

8.2. Please debate the results following the same order of the results section for the purposes of consistency. Start by addressing the factor structure, followed by the internal consistency, and ending with the association with demographic/clinical variables.

8.3. Between lines 336 and 343 (page 18), the argument is really confusing. “that is 26 and 20 factors for Awareness and ANSR”. Do the authors mean 2 factors with 26 and 20 items respectively? I had a hard time understand this argument so please rephrase this paragraph.

8.4. Although the statistical analysis is not my core expertise, the way the authors described their results from the MIA actually led me to think they are worse than they actually are. As I understood correctly, the authors would consider RMSEA values of .08 as satisfactory. Looking at Table 3 one can see that almost all values are bellow .08. Only the Strong and Strict steps had an RMSEA of .085 which is still quite satisfactory in my opinion. I think the authors should emphasize this more in both the Results and Discussion section.

8.5. The paragraph between lines 344 – 348 (page 18) addresses the main issue of this work, which is construct validity as I previously described. Although the translation process applied by the authors was outstanding, this work does not provide any data or analysis that allows them to truly state that the instrument is measuring body awareness and automatic reactivity. If the authors are not able to complete any sort of convergent/discriminant validity, this limitation should be explicitly debated here.

8.6. Although there is a clear need for convergent validity testing in my opinion, the authors should also clearly highlight that some expected associations with demographic variables were retained in the Italian sample, which suggests, as the authors frame it and very well, “solid robustness to cultural differences”. Thus, I would make a paragraph highlighting this right after the paragraph about the Measurement Invariance Analysis. Only after addressing this topic, I would talk about any other associations (physical activity, smoking, etc).

8.7. Please consider my comments above about correlation analysis for smoking and physical activities. If the authors want to debate this association, they should revise the statistical analysis as suggested.

8.8. After revising the statistical analysis for physical activity, if the author's results are retained, they should explore why their results are different from the previous study. The most likely reason, in my opinion, is due to the instruments used to assess interoceptive awareness in previous studies (reference 34 and 35). For instance, one of these studies uses the Multidimensional Assessment of Interoceptive Awareness which is a questionnaire that measures interoception with a very different framework from the BPQ.

8.9. Please improve the explanation about the association between body awareness, interoception, smoking, and interoception. I believe I understood what the authors meant, but the text is very unclear

8.10. As I argued in previous comments, I highly question the regression models computed to test the associations with pathology. Thus, the conclusion drawn between lines 379 – 389 should be removed if the authors do not address these previous issues (possibly replacing regression by t-tests), including describing how many subjects there were for each disease category.

8.11. At Line 385, Page 19 the authors state “a positive correlation has been found between ANSR supradiaphragmatic and psychiatric disease” but this is not described anywhere else, including the supplemental material. Furthermore, there were only 4 subjects with psychiatric disorders, which would not allow the authors to draw this conclusion. Please clarify this.

8.12. The Discussion section is clearly lacking a paragraph describing study limitations as there are many. I addressed several limitations across this review, and they should be included somewhere in the Discussion section, probably at the end.

8.13. In the Conclusion section, the authors may need to rephrase the sentence stating that this study supports the validity for measurement of body awareness and automatic reactivity if you do not address the issues about convergent validity.

9. Inconsistencies, References Issues, and Written Expression

Here I report the main inconsistencies, reference misplacement, and written expression problems that I detected, although there are several other similar issues that should be revised. I truly recommend a full document revision to make it easier for the reader.

9.1. Line 85, Page 4: The references for the BPQ and polyvagal theory are incorrect (20, 21, and 22).

Line 114, Page 5: Here the authors describe reference 1 as their guidelines for cross-cultural adaption. Then, in the Discussion section (line 348, page17) they state that the translation followed the WHO international guidelines (33). Furthermore, in the reference list, there is a paper about cross-cultural adaption guidelines (20) that is not included in the Methods sections. Please clarify this.

9.2. Line 137-138, Page 6: Not sure what the authors meant by “standard forms” and “consensus methodologies”

9.3. Line 141-142, Page 6: The authors should be clearer when they state they recorded data about physical activity, smoking habits, pathologies, and drug usage. For instance, participants were asked how many times they performed physical activity each week? Did they describe which activities or how long? Regarding smoking, it was just a yes or no question? Did the authors ask how many cigarettes? Regarding pathologies, was it an open-ended question and the type of pathology was categorized after data collection? The same for drug usage.

9.4. Line 155-157: The definition of construct validity is quite confusing. Do not know what the authors mean by “HR-PRO instrument”. Written expression is also not particularly clear.

9.5. Line 207, Page 10: Simply name this subheading “Internal consistency”

9.6. Line 212-213, Page 10: “However” does not really make sense in this sentence. It should be something like “We selected McDonald’s omega because ….”

9.7. Line 216, Page 10: I do not think that this subheading should be named “Reliability”. I would simply call it something like “Association of BPQ with demographic and clinical variables”.

9.8. Line 222, Page 10: Any reference to justify your cut-off point for floor/ceiling effects?

9.9. Line 235, Page 11: Please report the *p*-value for the r = .56 correlation.

9.10. Lines 271-273, Page 15: The reporting of confidence intervals for Cronbach’s Alpha is not very clear.

9.11. Line 331, Page 17: Incorrect reference

9.12. Lines 365-267, Page 18: Poorly written sentence

9.13. Line 375, page 18: "The linear regression confirmed all the correlations and highlighted new ones." Please be very careful with terminology regarding statistical findings. This sentence is incorrect. Also, please avoid using the term "correlated" when talking about regression results. Rather used "positively associated" or "negatively associated".

9.14. Table 1: Replace Height and Weight by BMI as this variable was the one used for regression models. I would remove Annual Income from this table as it is not used again for any analysis. As previously stated, I think it would be more important to report unemployment status for the reader to understand how many were university students, employed workers, etc. Finally, Sports should be replaced by Physical Activity as these concepts do have the same meaning. From what I understood, the authors assessed Physical Activity.

9.14. Tables 2 and 3: These tables are not mentioned in the text.

9.15. Table 2: I would add subtitles to the table to identify the reported statistics (e.g. P(>|z|)

9.16. Table 5: I did not feel that this table was necessary (just repeated data on the text).
