# Supplementary material for: Cross-cultural adaptation and psychometric properties of the Italian version of the Body Perception Questionnaire
Source: PLoS One. 2021 May 27;16(5):e0251838. doi: 10.1371/journal.pone.0251838 (PMC8158925; doi:10.1371/journal.pone.0251838)
Supplement: Attachment — Submitted filename: Rebuttal Letter.docx [file pone.0251838.s003.docx]

**Review of the manuscript**

**Manuscript number PONE-D-19-35072, entitled “Cross-cultural adaptation and validity of the Italian version of the Body Perception Questionnaire.”**

Dear editor,

Dear reviewers,

We greatly appreciate your readiness to have read our paper and to provide us with relevant feedback and useful suggestions to further improve the quality of our paper. A detailed description of all changes has been provided below.

For any further information, please do not hesitate to contact us.

**Editor**

1. Please ensure that your manuscript meets PLOS ONE's style requirements, including those for file naming. The PLOS ONE style templates can be found at

<https://journals.plos.org/plosone/s/file?id=wjVg/PLOSOne_formatting_sample_main_body.pdf>

and

<https://journals.plos.org/plosone/s/file?id=ba62/PLOSOne_formatting_sample_title_authors_affiliations.pdf>

**Response**: Thank you. Done.

2. We note you have included a table to which you do not refer in the text of your manuscript. Please ensure that you refer to Table 2 in your text; if accepted, production will need this reference to link the reader to the Table.

**Response:** Thank you. Done.

3. Please include captions for your Supporting Information files at the end of your manuscript, and update any in-text citations to match accordingly. Please see our Supporting Information guidelines for more information: http://journals.plos.org/plosone/s/supporting-information.

**Response:** Thank you. Done.

**Reviewer 1**

**1. Define study goals more clearly**

1.1. After reading the manuscript it seemed to me that this study had three main goals:

- Cross-cultural adaptation of the BPQ Short-Form for Italian.

- Examine the psychometric properties of the Italian BPQ Short-Form

- Examine the association between BPQ subscales and sociodemographic/clinical variables (e.g. age, BMI, smoking, diagnosis, medication intake, etc).

These goals should be stated at the end of the Introduction section and clearly noticed by the reader across the manuscript.

**Response:** Dear Reviewer, we appreciate your suggestion. The addition of main goals at the end of the introduction will make the reading of the whole paper more clear.

1.2. Clinimetric vs Psychometric: I know this is just a terminology issue, but I was surprised when the authors described their study as clinimetric. The terms are widely overlapping (De Vet et al. 2003 - 10.1016/j.jclinepi.2003.08.010) and some argue that clinimetrics is not a unique approach, but is rather a subset of psychometrics (Streiner, 2003 - 10.1016/j.jclinepi.2003.08.011). I believe that this term is not very frequently used nowadays (a quick search on PubMed records in the last 5 years confirmed this). I would suggest that the authors use the term psychometric study as it is much more widely used.

**Response:**  Dear Reviewer, the decision of using the term *clinimetrics* derived from De Vet et al. 2011 (*Measurement in medicine: a practical guide)* and from Feinstein 1987 (*Clinimetrics).* According to these authors we used *clinimetrics* as ‘measurement of clinical phenomena’. They focused on the construction of clinical indexes, and promoted the use of clinical expertise, rather than statistical techniques, to develop measurement instruments” (Feinstein 1987). We know that the terms are widely overlapping, and that *clinimetris* is not frequently used; for this reason, we accept your advice preferring *Psychometric.*

**Reviewer 1**

2. Improve the Introduction section: Although the introduction provides valuable information about body awareness and how this concept is linked to other phenoms (e.g. mental illness, management of chronic diseases), I still felt that this section was a little bit confusing and that some information was lacking.

**Response:** Dear Reviewer, thank you for your suggestions. We added the lacking informations and we structured the Introduction section as you suggest in the following points.

2.1. Although I know that the terminology in this field is quite heterogeneous, it would be important for me to clearly understand whether the authors consider body awareness and body perception similar constructs. The authors kind of state that at line 61 (page 3), but if the authors truly consider them as similar this should be explicitly stated at the start of the Introduction section. I actually believe it would be easier simply to talk about body awareness for consistency purposes, especially because it is one of the BPQ subscales.

**Response:** Thank you for your advice, we clarified better the constructs.

2.2. The introduction was mainly focused on defining and debating body awareness. This allows the reader to understand the body awareness domain, but not the autonomic nervous system reactivity subscale. Actually, I felt that this subscale was not evidently explained across the whole manuscript. The authors should clearly define both body awareness and automatic reactivity. I do not think this is an issue as the BPQ subscales are proposed to assess different domains and not a single unitary construct.

**Response:** Thank you! Done!

2.3. The authors introduced the concept of interoception and its association with body awareness and rightfully so in my opinion, as the BPQ is widely used as a measure for interoception in behavioral sciences. However, I believe the authors should further explore the most recently debated models about interoception when addressing this topic. This might seem unnecessary or not valuable for the main goals of this work, but in my opinion, this discussion is key when addressing the validity of this scale (especially for the body awareness subscale) and could help to interpret the results obtained by the authors. (more information below). If the authors feel that this information is not required in the Introduction, this should at least be debated in the Discussion section. Please check these three overviews about interoception measurement (the main ones that I know of in the last 5 years).

- Garfinkel et al. (2015) - 10.1016/j.biopsycho.2014.11.004

Argues that interoception encompasses three distinct dimensions (accuracy, sensibility, and awareness). According to these authors, the BPQ Body Awareness subscale measures interoceptive sensibility.

- Khalsa et al. (2017) – 10.1016/j.bpsc.2017.12.004

Proposed that interoceptive awareness encompasses a complex taxonomy (attention, detection, magnitude, discrimination, accuracy, sensibility, insight, self-report). This work also provides a lot of information about the link between interoception and mental health which I think is relevant for this manuscript.

- Murphy et al. (2019) – 10.3758/s13423-019-01632-7

Proposes a 2 x 2 factorial structure of interoception (factor 1: what is measured – accuracy vs. attention; factor 2: how is it measures – beliefs vs. performance). According to the authors, BPQ measures beliefs about interoceptive attention.

**Response** Thank you for your suggestion. The proposed literature has been added in the discussion

2.4. The authors decided to differentiate body awareness from somatosensory amplification. Although this is interesting from a conceptual standpoint, I am not sure if it really had a lot of valuable information as the authors do not address this concept in any other section.

**Response:** Dear Reviewer, as you rightly suggest, we have decided to omit the concept of somatosensory amplification to make the introductory section clearer.

2.5. I think that the structure of the introduction should be rethought for the purposes of clarity. The section is not very fluid. Reorganizing the several pieces while using some subheadings will likely make it substantially better and easier to follow. Thus, a possible structure would be (although there are other alternatives that may better suit the authors):

- Define body awareness (including its link to interoception) and autonomic reactivity;

- Describe the physiological circuits of body awareness and autonomic reactivity (including the polyvagal theory);

- Describe the importance of body awareness and autonomic reactivity for clinical disorders (e.g. mental health, other chronic disorders, etc) and for patient subjective reports;

- Highlight the lack of studies addressing the psychometric proprieties of measures related to body awareness and automatic reactivity. Introduce the BPQ.

- Study Goals

**Response:** Dear Reviewer, thank you for your suggestions. We improve the structure of the introduction as you request.

**Reviewer 1**

3. Organization of Methods Section

I do not think the Methods section is very well organized. The authors seem to go back and forth between the different stages of the study. Maybe try to follow something like:

- Participants (only describe participant recruitment, sample size, and participant characteristics)

- Measures

- BPQ: Start by describing the BPQ more clearly. This is not done in any section of the manuscript. For instance, the authors never mention that this questionnaire is completed through a 5-level Likert scale that rates frequency.

- Describe demographic and clinical data collected. End this section by provided information about the questionnaire application online

- Cross-Cultural Adaptation

- Statistical Procedures (provide an initial overview of statistics -info in lines 173-178 - before describing each specific step)

- Data Preparation

- Factor Analysis and Measurement Invariance Analysis

- Internal Consistency

- Association between BPQ and demographic/clinical variables

- Floor and ceiling effects

**Response:** Dear Reviewer, in order to improve the organization of this section, we accept your advices as possible to set “Methods” as you suggest. The description of BPQ can be found on the original validation paper. Therefore we would avoid to weightening the manuscript with those information.

**Reviewer 1**

4. Sample Characteristics

4.1. The authors state that they are assessing the BPQ validity in a sample of healthy subjects. However, I could easily argue that this not very accurate. First, all participants were contacted during clinical appointments with osteopaths, most of them probably due to some health-related condition. Secondly, around 30% of your sample reported some sort of disease and almost 25% reported medication intake. Although there is no specific information about the number of subjects per type of disease (this should also be added, at least in the supplemental materials), I think that considering this sample “healthy” is a little bit farfetched. I do not have any clear suggestions for the correct terminology that the authors should use, but clinical-sample or sample of health care services users would be more accurate in my opinion.

**Response:** Dear Reviewer, as well noted, we erroneously wrote in the abstract and in the introduction that our study was conducted on healthy subjects. As pointed out by your careful review, we have recruited subjects afferent to osteopathic care, and probably that may influence the selection of sample. If it is correct, we should expect a high presence of pathologies related to the osteopathic profession, which affect the musculoskeletal or neurological system. Musculoskeletal pathologies were not present in the questionnaire administered as they are very common. If we observe the number of participants by pathology - present in the table added on your suggestion - we observe that only about 5.5% of the total reports "other pathologies". We can assume that this percentage also contains musculoskeletal pathologies, which added to the percentage of subjects with neurological pathologies reaches about 8%. This fact does not make our sample composed of healthy subjects, but it reduces the influence of the selection mode. In conclusion we decided to substitute “healty subjects” with “Italian subjects”

4.2. The recruited sample is quite well-educated (40% with a university degree). Furthermore, about 30% of the participants had no income. These subjects with no income where mostly university students or just unemployed/retired? This should also be clear in my opinion as it can also strengthen the fact that this sample had above-average education levels. High education levels may be a possible source of bias that should be briefly introduced when describing study limitations.

**Response:** Dear Reviewer, observing the mean age (34) of our sample we can suppose that these subjects with no income were mostly university students or young degreed people looking for a job. This scenario is plausible with the Italian socio-economic situation.

**Reviewer 1**

5. Limitations of Online Surveys Should Be Addressed

5.1. The authors state the questionnaire was administered online, but there is not any more information about this. The authors should provide information on factors that are known to influence data quality in online surveys such as: average time of completion (probably not an issue in this study but should be reported); incentive for study completion (I guess there was not but this should be stated); missing values (most online studies do not have this issue but this should be stated as well); other factors the authors consider can influence data quality.

**Response**: Dear Reviewer, we really appreciate your suggestion and we added this information in our manuscript.

5.2. The authors did not describe any strategy to ensure data quality and exclude inadequate respondents. This may seem a minor issue, but there is evidence suggesting that careless respondents can range between 3 to 60% in self-report studies. Nowadays there are several methods to address this issue (for more information check Curran 2016 - 10.1016/j.jesp.2015.07.006 - and Niessen et al. 2016 - 10.1016/j.jrp.2016.04.010), but I recommend that the authors at least exclude subjects based on response time. Niessen et al. (2016) reported that response time was one of the most effective methods to detect careless responding. When subjects take more time to complete the questionnaire, data quality is better and there is less probability of careless responding. The authors should establish a cut off criteria to exclude subjects based on response time. There are also several suggestions about which criteria to use (e.g. three-second-per-item rule, exclude subjects 3 standard deviations below the average response time, etc) and the authors should select the criteria they feel it is more accurate (and justify it with previous studies). If the authors have not recorded response times, please look at other statistical procedures (e.g. multivariate outlier analysis). The authors may also check if there were answers submitted from duplicate IP addresses, although in this case, this seems to be highly unlikely.

**Response** : Dear Reviewer, unfortunately we did not recorded time of BPQ compilation, the use of Google Form returned us only the timestamp when sending reply.

5.3. Finally, the limitations of online surveys should be addressed in the Discussion section. Although participants were directly contacted, I am assuming that only subjects with internet access could participate (unless the authors had any solution to address this). This introduces a slight bias that should be referred (for instance, older participants are more likely to have no internet access).

**Response**: Dear Reviewer, acute observation. Of sure internet connection was needed, but the direct contact with the recrutator, often in a Wi-Fi open zone, may eased the subministation of BPQ.

**Reviewer 1**

6. Issues with Psychometric Analysis

Although the psychometric analysis is quite interesting, I would like to clarify several issues with the authors:

6.1. Construct Validity: The authors argue that they will achieve construct validity through hypothesis testing and propose some hypotheses in the Methods sections without providing previous evidence for them. This evidence should be quite strong and from several sources ideally. I would like the authors to argue whether there is clear evidence about the relationship between body awareness and automatic reactivity with age, but more importantly to the other proposed variables such as smoking, physical activity, BMI, and educational level. Furthermore, why did the authors did not include gender in this hypothesis testing as they address it on the Results and the Discussion section? Regardless, I do not believe that this strategy is sufficient for construct validity, especially pertaining to body awareness as there are so many authors arguing how interoception encompasses several measurement dimensions (as described above). This is the main issue of this work in my opinion. For me, the only alternative to accomplish this would be to examine convergent and/or discriminant validity. For instance, Cabrera et al. (2017) used the Stress Reactivity Index and SomatoSensory Amplification Scale for convergent validity. As several authors also consider that the body awareness subscale assesses interoceptive sensibility or interoceptive attention, another alternative would be using interoception questionnaires to test for convergent/discriminant validity. Alexithymia could also be measured together with interoception as a recent meta-analysis from Trevisan et al. (2019) found that the BPQ was positively associated with alexithymia, although other measures of interoception were negatively associated (10.1037/abn0000454). Finally, depression and anxiety scales could also be an interesting alternative as they have been differentially associated with interoceptive scales, including the BPQ (e.g. Murphy et al. 2019 - 10.1177/1747021819879826). There should also be several other interesting alternatives for convergent or discriminant validity regarding the automatic reactivity subscale. Basically, if the authors want to dramatically improve their work and provide clear evidence that the Italian BPQ is truly measuring body awareness and automatic reactivity is to complete another study with a smaller sample size (I would estimate about 100 although a power analysis would be needed). If this is not possible for the authors, then they should acknowledge that this work is extremely limited to provide evidence for construct validity-and provide recommendations for future studies to test this.

**Response:** Thank you for your very insightful comment. The construct validity was based on the COSMIN framework, which provided the following elements for the construct validity (including structural validity, hypotheses testing, and cross‐cultural validity - <https://www.cosmin.nl/wp-content/uploads/COSMIN-methodology-for-content-validity-user-manual-v1.pdf> ). So following this framework, we believe that the methods used fulfilled the COSMIN criteria.

Although we recognised the importance of comparing the BPQ with other measurements (which might be referred to as convergent or divergent validity), due to time constraints to resubmit the manuscript, we preferred to include those elements suggested in the limitations of the study.

6.2. Although it is not my core expertise, I have some questions to be clarified/improved by the authors regarding the Confirmatory Factor Analysis (CFA) and Measurement invariance analysis (MIA). First, the description of the CFA procedures in the Methods section seems too broad. Further details should be given to the reader (e.g. type of matrix used, estimation procedure, etc), at least in Supplemental material. Second, the indicators chosen for Table 2 regarding the CFA are not very usual. If I understood correctly, the estimates are the unstandardized factor loadings, with their corresponding standard errors. Why did the authors select not to report the standardized loadings (more common) as it was done for instance by Cabrera et al. (2017)? Is it actually necessary to report “P(>|z|)| as the values are similar for each item? For more information about reporting a CFA I advise the authors to consult the work from Jackson et al. 2009 - 10.1037/a0014694. Third, I wanted to know why the authors selected to report the Chi-Square Statistics for the MIA but not for the CFA. Conversely, why did the authors report the TLI for the CFA but not for the MIA. This should be explained to the reader.

**Response**: Dear Reviewer, thank you for your comments. The decision of the indicators is referred to Hu and Bentler “Cutoff criteria for fit indexes in covariance structure analysis: Conventional criteria versus new alternatives. Structural Equation Modeling. 1999;6(1):1–55”. The P(>|z|) = 0.000 shows the significance of the CFA on the model, we think that is important for the reader. The choice to report these values for MIA and CFA are to be found in the sources of Hu and Bentler (1999) and Putnick and Bornstein “Measurement invariance conventions and reporting: The state of the art and future directions for psychological research. Vol. 41, Developmental Review. Mosby Inc.; 2016. p. 71–90”.

6.3. In lines 181-183 the authors state that they dichotomized ratings as it was done on previous psychometric analysis of the BPQ (I believe the authors are refereeing to the paper from Cabrera et al. 2017, although the reference numbers seem incorrect). This is a valid argument, however, in the work from Cabrera et al. 2017 the authors actually decided to dichotomize the items as the exploratory factor analysis using full item distributions resulted in a large number of factors and loadings with complex factors. Did the authors perform any sort of factor analysis for the full-item distribution in this proposed Italian version? If so, this should be addressed somewhere (even if it is in the supplements). If not, I believe it will be important for the authors to complete this analysis. If the questionnaire was applied with a 5-point Likert scale, I believe that this should not be simply left out because it can provide valuable information for future studies.

**Response**: Dear Reviewer, we really appreciate your careful comment. In this study we decide to follow the same process of validation used from Cabrera 2017 that dichotomized data to perform EFA. With the same their reason -large number of factors and loadings with complex factors- we performed CFA only on the dichotomized data. While the BPQ is on a 5-point Liker type scale, but we think was important to follow the procedures of previous papers.

6.4. Item “I feel like vomiting”: The authors only address the fact that this item was included on both reactivity subdomains in the Descriptive Statistics heading. This should be already explained in the Methods sections. Also, I think the authors should justify why did they select to keep the item on both subdomains In the work of Cabrera et al. (2017) the supra- and subdiaphragmatic reactivity loadings were a problem in the exploratory factor analysis of the Spanish sample. However, they also described that in the CFA factor loading of the American sample the item was associated more strongly with subdiaphragmatic reactivity. Thus, I think the authors should clearly explain why this item was retained on both subdomains in the Italian version.

**Response:** Dear Reviewer, we really appreciate your constructive revision. As you suppose we select to keep the item in both subdomains according to Cabrera et al 2017. In order to clarify this choice we decided to explicit it in the Methods section.

6.5. The authors did not report the correlations between the supra- and sub-diaphragmatic reactivity scores with the body awareness score. This should be added.

**Response:** Thank you for your suggestion, we added the correlation between reactivity scores and awareness score.

6.6. In the internal consistency subheading, the authors should also report the confidence intervals for McDonald’s omega.

**Response:** Thank you. Done.

**Reviewer 1**

7. Testing the association between BPQ and demographic/clinical variables.

7.1. In the methods section (lines 217-220, page 10) the authors should state that subscale scores were calculated based on dichotomized items. Here the authors should also briefly describe which regression models were tested (how many, dependent, and independent variables).

Response: Thank you. Done.

7.2. I would also like to know why the authors decided to use both Pearson and Kendall correlations, which are parametric and non-parametric, respectively. Was that the reason? If so, did the authors did any kind of assumption testing before deciding to select both?

**Response:**Dear Reviewer, thank you for your comment. We decided to perform both Pearson and Kendall correlation not considering parametric and non-parametric features of the tests. Kendall rank test is more statistical correct to assess correlation in Likert scales (Arndt et al. 1999). However, Kendall tau seems to be stronger in a small/moderate sample, therefore we decided to compare it with the Pearson test.

7.3. In the results section, the authors describe the association between the BPQ subscales and each demographic/clinical variable. This is quite confusing for the reader. I do not understand if they prepared a regression model for each predictor or if they had a complex model with all the predictors. For the purpose of clarity, I think the authors should organize this section by first describing the significant correlations found (based on Table 6) and only them report the results of regression models (assuming the authors had one model with all predictors).

\

**Response:** Thank you! Done

7.4. Table 6 should be presented as a correlation matrix. The way it is presented now is quite confusing. Statistically significant correlations should be highlighted in some way.

**Response:** Thank you! Done!

7.5. The authors reported correlations for (supposedly) categorical variables such as Smoking and Physical Activity. Computing correlations is not very advisable here in my opinion. The more adequate statistical approach would be to compare Smoker vs. Non-Smokers or Physical Active vs. Inactive using independent samples t-tests.

**Response**: Dear Reviewer, we complete the analysis suggested and we add them to the correlation computed.

7.6. I think that Figure 1 is not necessary as this correlation could be merely reported in Table 6 and in the text.

**Response:** Dear Reviewer, thank you for your comment, we added the Fig. 1 to visually represent the the correlation, but if you think it superfluous, we remove it.

7.7. I have several issues with the linear models sent in the supplementary material, which are actually described in the manuscript. First, I do not understand why the authors computed a regression model with the BPQ total score. The authors do not talk about a BPQ total score across the whole manuscript and I do not think the BPQ was designed to have a total score. At line 300, page 15 the authors also address this model. This analysis should not be reported in my opinion. Secondly, the authors presented linear models with more than 20 predictors. I do not think that the sample size is adequate for this analysis. Third, the authors do not report how many subjects have each pathology. For instance, if you include psychiatric disorders as a predictor, there are only 4 subjects with a diagnosis. This cannot provide valuable insights in a regression model. This issue is probably true for other diagnoses, but the authors must clarify how many subjects are in each type of pathology. If the authors are interested in exploring the association between BPQ and diagnosis, maybe it would be easier to perform independent group t-tests for each diagnostic group (only for groups with significant sample size) and report mean and standard deviation for each group. You can even control the p-value for multiple comparisons. Mann–Whitney U tests may also be used if the sample size is reduced and/or normality assumption is not met

**Response:** Dear Reviewer, thank you for your comment. In order to your suggestion we remove the results of the BPQ total score.We also added the numbers of each pathology in supplementary materials. However, in the regression model used pathologies are considered as an independent factor, which is weighted within the model, for this reason we think that the use of these analyses is adequate.

7.8. At line 311, Page 16 the authors describe an ANOVA analysis that was never mentioned in the Methods sections.

**Response**: Thank you! Done

**Reviewer 1**

8. Enhance the Discussion section

8.1. The first paragraph (lines 321-323, page 16) should reinforce the 3 main goals that I argued at the beginning of my review. As it is now written it seems to empathize only internal consistency which is only a part of the authors work.

**Response:** thank you for your comment. Done.

8.2. Please debate the results following the same order of the results section for the purposes of consistency. Start by addressing the factor structure, followed by the internal consistency, and ending with the association with demographic/clinical variables.

**Response:** Thank you for your comment. Done

8.3. Between lines 336 and 343 (page 18), the argument is really confusing. “that is 26 and 20 factors for Awareness and ANSR”. Do the authors mean 2 factors with 26 and 20 items respectively? I had a hard time understand this argument so please rephrase this paragraph.

**Response:** Thank you for your comment. The paragraph has been rephrased.

8.4. Although the statistical analysis is not my core expertise, the way the authors described their results from the MIA actually led me to think they are worse than they actually are. As I understood correctly, the authors would consider RMSEA values of .08 as satisfactory. Looking at Table 3 one can see that almost all values are bellow .08. Only the Strong and Strict steps had an RMSEA of .085 which is still quite satisfactory in my opinion. I think the authors should emphasize this more in both the Results and Discussion section.

**Response:** Thank you! We add the explanation of why we intend the MIA as partial.

8.5. The paragraph between lines 344 – 348 (page 18) addresses the main issue of this work, which is construct validity as I previously described. Although the translation process applied by the authors was outstanding, this work does not provide any data or analysis that allows them to truly state that the instrument is measuring body awareness and automatic reactivity. If the authors are not able to complete any sort of convergent/discriminant validity, this limitation should be explicitly debated here.

**Response:** Thank you for your comment. Unfortunately we are not able to provide the requested analysis. Therefore, we added the following sentence: “*Moreover, since it was not planned a convergent/discriminant validity testing it might be difficult to assess to what extent the Italian version of the BPQ measures body awareness and automatic reactivity*.”

8.6. Although there is a clear need for convergent validity testing in my opinion, the authors should also clearly highlight that some expected associations with demographic variables were retained in the Italian sample, which suggests, as the authors frame it and very well, “solid robustness to cultural differences”. Thus, I would make a paragraph highlighting this right after the paragraph about the Measurement Invariance Analysis. Only after addressing this topic, I would talk about any other associations (physical activity, smoking, etc).

**Response:** Thank you for your suggestion. Done. We added the recall to the figure 1 to ease the comprehension

8.7. Please consider my comments above about correlation analysis for smoking and physical activities. If the authors want to debate this association, they should revise the statistical analysis as suggested.

**Response**: Thank you. Done.

8.8. After revising the statistical analysis for physical activity, if the author's results are retained, they should explore why their results are different from the previous study. The most likely reason, in my opinion, is due to the instruments used to assess interoceptive awareness in previous studies (reference 34 and 35). For instance, one of these studies uses the Multidimensional Assessment of Interoceptive Awareness which is a questionnaire that measures interoception with a very different framework from the BPQ.

**Response:** Thank you for your comment. We added the literature you suggested in a comment above to discuss the tool/related difference.

8.9. Please improve the explanation about the association between body awareness, interoception, smoking, and interoception. I believe I understood what the authors meant, but the text is very unclear

**Response:** Thank you for the comment. The sentence has been rephrased as follow: “ *A possible explanation for the correlation is proposed by Naqvi & Bechara (41): the interpretation framework between the awareness of the interoception and smoking takes into account that the insula - which can be affected by nicotine - makesing the interoceptive information available to conscious awareness*.”

8.10. As I argued in previous comments, I highly question the regression models computed to test the associations with pathology. Thus, the conclusion drawn between lines 379 – 389 should be removed if the authors do not address these previous issues (possibly replacing regression by t-tests), including describing how many subjects there were for each disease category.

**Response:** Again, thank you for your comment. As above mentioned we added the numbers of each pathology in order to have more comprehensive information. However, we believe that the regression model used can be considered adequate as pathologies are considered as an independent factor, which is weighted within the model. Besides, as the total sample is sufficient for justifying the multivariate analysis based on all the factors included, we believe that the Student t-tests might produce misleading and less informative results.

8.11. At Line 385, Page 19 the authors state “a positive correlation has been found between ANSR supradiaphragmatic and psychiatric disease” but this is not described anywhere else, including the supplemental material. Furthermore, there were only 4 subjects with psychiatric disorders, which would not allow the authors to draw this conclusion. Please clarify this.

**Response:** Thank you for your accurate reading. We corrected the wrong sentence adding the following sentence: “*Our findings showed a positive correlation between ANSR Subdiaphragmatic subscale and total score and the use of psychoactive drugs.*”

8.12. The Discussion section is clearly lacking a paragraph describing study limitations as there are many. I addressed several limitations across this review, and they should be included somewhere in the Discussion section, probably at the end.

**Response:** Thank you for your suggestion. We added a sentence about limitations after each section of the discussion to keep it contextualized as much as possible.

8.13. In the Conclusion section, the authors may need to rephrase the sentence stating that this study supports the validity for measurement of body awareness and automatic reactivity if you do not address the issues about convergent validity.

**Response:** Please refer to the comments 6.1

**Reviewer 1**

9. Inconsistencies, References Issues, and Written Expression

Here I report the main inconsistencies, reference misplacement, and written expression problems that I detected, although there are several other similar issues that should be revised. I truly recommend a full document revision to make it easier for the reader.

9.1. Line 85, Page 4: The references for the BPQ and polyvagal theory are incorrect (20, 21, and 22).

**Response:** Thank you. Done.

Line 114, Page 5: Here the authors describe reference 1 as their guidelines for cross-cultural adaption. Then, in the Discussion section (line 348, page17) they state that the translation followed the WHO international guidelines (33). Furthermore, in the reference list, there is a paper about cross-cultural adaption guidelines (20) that is not included in the Methods sections. Please clarify this.

**Response:** Thank you for your comment. Reference 1 refers to the statistical and conceptual methods. Reference 33 refers to the sole translation process. Reference 20 has been changed. However, the numbers of the references will change in the present version because we added some new ones and changed the order of others.

9.2. Line 137-138, Page 6: Not sure what the authors meant by “standard forms” and “consensus methodologies”

Response: Thank you for your comment. It has been rephrased.

9.3. Line 141-142, Page 6: The authors should be clearer when they state they recorded data about physical activity, smoking habits, pathologies, and drug usage. For instance, participants were asked how many times they performed physical activity each week? Did they describe which activities or how long? Regarding smoking, it was just a yes or no question? Did the authors ask how many cigarettes? Regarding pathologies, was it an open-ended question and the type of pathology was categorized after data collection? The same for drug usage.

Response: Thank you for your comment. Most of the answers to your questions can be found in Table 1. Since the manuscript is almost around 5000 words we would avoid to add more in text content to avoid making it too heavy.

9.4. Line 155-157: The definition of construct validity is quite confusing. Do not know what the authors mean by “HR-PRO instrument”. Written expression is also not particularly clear.

Response: Thank you, “HR-PRO instrument” means “Health Related Patient-Reported Outcome”. We added the extended expression in the manuscript.

9.5. Line 207, Page 10: Simply name this subheading “Internal consistency”

**Response**: Thank you! Done!

9.6. Line 212-213, Page 10: “However” does not really make sense in this sentence. It should be something like “We selected McDonald’s omega because ….”

Response: Thank you. Done.

9.7. Line 216, Page 10: I do not think that this subheading should be named “Reliability”. I would simply call it something like “Association of BPQ with demographic and clinical variables”.

**Response**: Thank you. Done.

9.8. Line 222, Page 10: Any reference to justify your cut-off point for floor/ceiling effects?

**Response**: Dear Reviewer, we add this reference : ” Terwee et all. (2007). Quality criteria were proposed for measurement properties of health status questionnaires. *Journal of clinical epidemiology*, *60*(1), 34-42.”

9.9. Line 235, Page 11: Please report the p-value for the r = .56 correlation.

**Response**: Thank you! Done!

9.10. Lines 271-273, Page 15: The reporting of confidence intervals for Cronbach’s Alpha is not very clear.

Response: Thank you. Done.

9.11. Line 331, Page 17: Incorrect reference

Response: Thank you. Done.

9.12. Lines 365-267, Page 18: Poorly written sentence

Response: Thank you. Done.

9.13. Line 375, page 18: "The linear regression confirmed all the correlations and highlighted new ones." Please be very careful with terminology regarding statistical findings. This sentence is incorrect. Also, please avoid using the term "correlated" when talking about regression results. Rather used "positively associated" or "negatively associated".

Response: Thank you. Done.

9.14. Table 1: Replace Height and Weight by BMI as this variable was the one used for regression models. I would remove Annual Income from this table as it is not used again for any analysis. As previously stated, I think it would be more important to report unemployment status for the reader to understand how many were university students, employed workers, etc. Finally, Sports should be replaced by Physical Activity as these concepts do have the same meaning. From what I understood, the authors assessed Physical Activity.

**Response:** Thank you! Done!

9.14. Tables 2 and 3: These tables are not mentioned in the text.

**Response**: Thank you! Done!

9.15. Table 2: I would add subtitles to the table to identify the reported statistics (e.g. P(>|z|)

**Response**: Thank you! Done!

9.16. Table 5: I did not feel that this table was necessary (just repeated data on the text).

**Response**: Thank you. Table deleted.

Sincerely,

The authors
