# Supplementary material for: Cross-cultural adaptation and psychometric properties of the Italian version of the Body Perception Questionnaire
Source: PLoS One. 2021 May 27;16(5):e0251838. doi: 10.1371/journal.pone.0251838 (PMC8158925; doi:10.1371/journal.pone.0251838)
Supplement: Attachment — Submitted filename: Rebuttal Letter.docx [file pone.0251838.s004.docx]

**Review of the manuscript**

**Manuscript number PONE-D-19-35072, entitled “Cross-cultural adaptation and validity of the Italian version of the Body Perception Questionnaire.”**

Dear editor,

Dear reviewers,

We greatly appreciate your readiness to have read our paper and to provide us with relevant feedback and useful suggestions to further improve the quality of our paper. A detailed description of all changes has been provided below.

For any further information, please do not hesitate to contact us.

**Reviewer 1**

**- BPQ Total Score**

In their response letter the authors state that they excluded the results from the BPQ Total Score. However, they still address the total score in several sections of the manuscript:

Page 10: “The Cronbach’s alpha and McDonald’s omega were calculated for the total scale and after removing each item to evaluate this measurement property.”

Page 10: “In the four linear regressions we set, each one with the respective subscales of the BPQ (Awareness ANSR Supra and Sub diaphragmatic and BPQ total score) …”

Page 16: “In these cases we identified a positive significant correlation with BPQ total score and the presence of Cranio-Facial Pathologies …”.

Page 20: “Our findings showed a positive correlation between ANSR Subdiaphragmatic subscale and total score and the use of psychoactive drugs.”

The authors should remove all references to the total score if they truly excluded these analysis from their work.

**Response:** Thank you for your suggestion. All the sentences referring to the total score have been removed as suggested.

**- Binary Scoring System**

In my first revisions, I asked why the authors decided to use the binary scoring system for the factor analysis. The authors argued decided that they decide to replicate the previous work. However, I still think the authors should provide alternative results for the full-scoring system, even if these results are displayed in the supplementary materials. The full-scoring system for the BPQ provides much more sensitivity for individual differences and should be used rather than the binary scoring system.

Thus, the authors should at least present an EFA with the full-scoring ratings. This is valuable information for the field as it would allow to understand if in the Italian the full-scoring rating also leads to a large number of factors and loadings with complex factors.

**Response:** Thank you for your comment. We have addressed this issue.

**- Statistical Analysis and Reporting**

1) The CFA procedures described in the Methods section is still quite incomplete. Further details should be given to the reader (e.g. type of matrix used, estimation procedure, are factors allowed to correlate or not, etc), at least in Supplemental materials.

2) Correlations with dichotomous variables: Even after the first revisions, the authors still describe and discuss correlations between scores and dichotomous variables like Smoking, Physical Activity or Drug Usage. As I argued before, correlations are not well suited for dichotomous variables. Correlations should be used with continuous variables such as Age or BMI. For dichotomous variables (e.g. Smoking, Physical Activity, Drug Usage, etc), the authors should only report independent samples t-tests comparing both groups (e.g. Drug Users vs. Non-Drug Users).

3) Regression models: I still do not clearly understand which regression models were implemented. More particularly, it is not clear which predictors were used. It seems that age, gender, physical activity, pathologies and drugs were used in the models presented in the supplementary tables. Smoking and BMI were not included in these models, apparently. Why? And psychiatric disorders were not included as well! This predictor is an important clinical outcome. Why was is not included?

**Response:** Thank you for your suggestion, we conducted and reported the new analysis that you request.

More importantly, I cannot agree with the authors argument for conducting the models with all the pathologies and drugs as predictors. First, the authors presented linear models with more than 20 predictors. The sample size is not adequate for that many predictors. Secondly, there are predictors which do not have a sufficient amount of subjects per category to allow them to be included in the model.

For instance, there is only 1 subject that reported Immunosuppressor drug use. How can this be a predictor in the model? The same for cancer (n=2) and haematological disorders(n=3)? I do not understand the argument that “used pathologies are considered as an independent factor, which is weighted within the model”. The problem in the analysis is that these models include several dichotomous predictors which lack an adequate number of subjects per category. This is inadequate in my opinion, regardless of the independent factor argument.

4) Reporting of statistical results is not very adequate and does not follow any guidelines. The authors should at least report data for statistical tests using the APA guidelines.

**Response:** Thank you for your suggestion. To overcome the problem of the appropriateness of models we decided to conduct linear regression using a stepwise method to select predictors.

**- Limitations and Recommendations for future studies**

The authors stated that they added a sentence about limitation after each discussion section. However, I still feel that there the manuscript lacks a clear paragraph describing limitations and recommendations for future studies. For instance, the authors do not debate the limitations of their sampling procedures. Collecting data only from subjects looking for osteopathic care is a limitation of this study. Furthermore, this sample is quite well-educated (40% with a university degree), which further limits the conclusions of this work. Also, the authors could also provide clear suggestions for future studies that would allow for convergent validity.

**Response:** Thank you for your comment. A dedicated section has been added.

**- Written Expression and Citations**

Written expression is still quite flawed across the document. Several sentences are extremely hard to understand. To be considered for publication, this manuscript should be revised by someone fluent in English writing. There are also still a few issues with citation. For instance, the work form Cabrera et al. (1) is cited across the text in sentences where it is not adequate in my opinion. This should also be addressed.

**Response:**  Thanks for your comment. The manuscript has been revised by native English speakers.

**Introduction**

This section has improved significantly since the last review. The constructs (and how they are linked) are more plainly defined. The text is also much more readable and clear for the reader. There is still one minor issue to be addressed.

- Page 3, 2nd paragraph: In my opinion, if body awareness equates to body perception in the authors’ opinion, this concept should be introduced in the first paragraph: “…. Clustered into a construct called body awareness (also known as body perception)”.

Furthermore, the definition of body awareness provided in the 2nd paragraph should be moved somewhere in the first paragraph. It does not make much sense to present the definition only in the 2nd paragraph as the construct was already introduced to the reader in the previous paragraph.

**Response:** Thank you for your comment. We added the specifications as per your suggestion. We feel that, following the flow of the discourse, there is no need to change the order of the presented topics.

**Methods**

Page 5

- In authors reply to my original review, they state: “… we have recruited subjects afferent to osteopathic care, and probably that may influence the selection of sample. If it is correct, we should expect a high presence of pathologies related to the osteopathic profession, which affect the musculoskeletal or neurological system.” – I believe this issue should be addressed in the Participants heading.

**Response:** Thank you for your comment. Only 2% declared to have neurologic disorders, and we have no data concerning the musculoskeletal conditions of participants (which would fall eventually into “others disorders” that account for 5%). So we don’t feel we can support that statement.

- “The total number of participants was calculated taking into account at least 10 participants for each item of the questionnaire”. Unusual phrasing to explain sample size calculation. Please improve.

**Response:** Thanks for your comment. The manuscript has been revised by native English speakers.

- The questionnaires were completed “in the presence of the osteopath” but it still not clear where. Private practices of each osteopath?

**Response:** Thank you for your comment. “In their private practice” has been added to the sentence.

- “The use of this compilation method …”. Not sure what the authors means by “compilation method”.

**Response:** Thank you for your comment. The compilation method refers to the google form which was set to “all mandatory responses” in order to avoid missing answers.

Page 6

- Incomplete information on sociodemographic and clinical variables. For instance, regarding physical activity you should at least state that you asked whether subjects performed physical activity ≥2 times/week. Regarding smoking, it should be clear that it was a yes and no question. “Smoking habits” leads readers to wonder whether you asked how many cigarettes per day or per week.

**Response:** Thank you for your comment. A specification of the timing of physical activity has been added to the table. 1. The “smoker” question did not ask for the number of cigarettes.

- “It has demonstrated strong psychometric properties and a consistent factor structure across multiple languages”. – This is somewhat of an overstatement. To my knowledge, the BPQ was only formally valeted in English and Spanish.

**Response:** Thank you for your comment. In this website ([https://www.stephenporges.com/body-scales**https://www.stephenporges.com/body-scales**](https://www.stephenporges.com/body-scales)**)** it’s possible to find all the languages in which the BPQ has been translated.

- The BPQ description is still far too incomplete for a validation paper in my opinion. Although some information can be found in the Results section, I believe that this should be presented together with the scale description. The authors should describe how many items are included in each domain. Also, there is an item that is included in both the supradiaphragmatic reactivity and the subdiaphragmatic reactivity domains. This should be transparent for the reader. It should also be clear that separate scores are completed for the Body Awareness and Autonomic Reactivity domains (the BPQ manual does not even address a total score).

**Response:** Thank you for your suggestion. Done.

Page 7

- “The process of cross-cultural adaptation followed established guidelines 1.” – This citation does not seem right as the work from Cabrera does not provide any sort of guidelines for cross-cultural adaptation.

**Response:** Thank you for your suggestion. The sentence has been rephrased.

Page 8

- Again, citation number 1 (Cabrera et al.) seem to be inadequately used to explain structural validity and construct validity. Please use adequate citations to justify your rationale for psychometric analysis.

**Response:** Thank you for your comment. As we changed the introduction sentence (previous comment) now it’s clear that we refer to cabrera statistical analysis since we followed the same procedure of that cross-cultural validation of the same questionnaire.

Page 9

- “As suggested by the Consensus-based Standards for the selection of health Measurement Instruments (COSMIN) initiative 23 and the International Society for Quality of Life Research (ISOQOL) 22” – This sentence seems incomplete.

**Response:** thank you for your comment. The sentence has been rephrased.

- “The hypotheses were that the age was normally distributed with respect to the Awareness subscale values ….” – I could not understand what the authors meant with this sentence.

**Response:** Thanks for your comment. The manuscript has been revised by native English speakers.

- “Furthermore, we hypothesized that Smoking habits, BMI and educational level with the Awareness Subscale values.” – This sentence seems incomplete or incorrect.

**Response:** thank you for your comment. The sentence has been rephrased.

- “CFA was performed considering the subscales of BPQ, the Awareness and ANSR Supra and Sub-diaphragmatic.” – It is not clear that you conducted two separate CFA: 1) a one-factor solution for the Body Awareness domain; 2) a two-factor solution for the Autonomic Reactivity domain. Please improve this.

**Response:** Thank you for your comment. The sentence has been improved following your suggestion.

Page 10

- “The Cronbach’s alpha and McDonald’s omega were calculated for the total scale and after removing each item to evaluate this measurement property. We evaluated the internal consistency related to BPQ Awareness, BPQ ANSR total, and in the supra/subdiaphragmatic subscales.” This is extremely confusing. First, the authors state that they computed the internal consistency for the total scale, which does not make much sense as there is no formal recommendation to use the total score for the BPQ. Then, I do not understand what the authors meant when they state “… after removing each item to evaluate this measurement property.”. Please clarify.

**Response:** Thank you for your comment. The “total score” has been removed and the sentence has been rephrased.

- I think the authors should describe cut-off scores to classify internal consistency scores.

**Response:**Thank you for your comment. Done.

- “The study of the correlations among BPQ scores and demographic characteristics was performed using Linear Regression Model, and estimating Kendall and Pearson’s indicators.” – Extremely unusual written expression. Please improve.

**Response:** Thanks for your comment. The manuscript has been revised by native English speakers.

- “Studying Smoke and Physical activity variables, we computed t-test between smokers and no smokers, and between active and inactive subjects, in order to assess differences in Awareness and ANSR subscales” – Somewhat confusing sentence. Please improve.

**Response:** Thanks for your comment. The manuscript has been revised by native English speakers.

- “In the four linear regressions we set, each one with the respective subscales of the BPQ (Awareness ANSR Supra and Sub diaphragmatic and BPQ total score)” – Still wondering why a regression model was computed for the BPQ Total Score. What does this BPQ Total Score measure actually, according to the authors? For me it seems that the BPQ subscales address different constructs (although they can be related with each other). So, if the authors use the BPQ total score they should clearly define what this total score represents.

**Response:** Thank you for your comment. All the “total score” related sentences have been removed.

“ … as the dependent variables and the demographic and clinical characteristics of the sample as independent variables.” – It is still not clear which variables were included as predictors. This is not clearly described on any part of the manuscript.

**Response:** Thanlìk you! Done!

Page 11

- “Floor and ceiling effects were considered to be present if ≥ 15% of the patients reported the lowest (0) or highest (47) possible BPQ score” – The BPQ Short Form only was 46 items. So the total score should only be 46, using the binary scoring system. If the authors considered the max score of 47, this is because they probably included item #41 (“I feel like vomiting”) twice, because this item is included on both the Supra and Sub Diaphragmatic domains. This would be incorrect in my opinion. Regardless, I do not think the authors should even consider the total score for analysis. However, if they decide to do so, this issue should be addressed.

**Response:** Thank you for your comment, we added an explanation.

**Results**

Page 14

- “The BPQ‐ SF was scored by adding the dichotomized responses (0 = never, 1 = occasionally or more often) in accord to the factor structure described above, with “I feel like vomiting” included in both reactivity scales (supra and subdiaphragmatic subscales).” – In my opinion, this information should be in the Methods section, rather than here.

**Response:** Thank you for your comment. Following your previous comment we added the information also in the methods.

Page 15, 16 and 17

I still find the description of the results of the section “Relation to demographic variables” quite confusing, for several reasons:

1. Why did you retain the correlation between Physical Activity and Smoking Habits? This variables are dichotomous correct? Thus, only the t-test should be used, not the correlation analysis.

**Response:** Thank you for your comment. We reorganized this section adding the analysis suggested.

2. The authors stated that they removed the analysis for the Total score in their reply to the first revision. However, they still report the regression model for the total score. Please clarify this.

**Response:** Thank you for your comment. All the “total score” related sentences have been removed.

3. The results are presented without any clear structure and it very hard to follow them. For instance, I still don’t understand clearly which variables were included in the regression models. In the results section, may be it would be easier to report the correlation, t-tests and ANOVA first. Them, prepare one short paragraph for each regression model, so that the reader can clearly understand the results.

**Response:**Thank you for your comment. We reorganized this section adding the analysis suggested.

4. Statistical reporting is quite poor. The authors should consider the APA guidelines for reporting their results.

**Response:**Thank you for your comment. Done.

Page 16

- I still think that Figure 1 should be removed. It’s not useful to interpret the study results.

**Response:** Thank you for your comment. Done.

Page 17

- “Floor or Ceiling effects were not present in our sample considering the ≥ 15% of the patients

reported the lowest (0) or highest (47) possible BPQ score” – Improve written expression.

**Response:** Thanks for your comment. The manuscript has been revised by native English speakers.

**Discussion**

Page 18

- “For these reasons, we argue that the results of the MIA influence only partially the cross-cultural adaptation and the validity of the Italian version of the BPQ-SF” – Improve written expression

**Response:** Thanks for your comment. The manuscript has been revised by native English speakers.

- “The internal consistency showed to be very high for both subscales of the BPQ, and for the two subscales of ANSR (Table 5).” – To make this statement the authors should have defined cut-off criteria for the internal consistency measures. Please address this.

**Response:** Thank you. Done.

- “Moreover, since it was not planned a convergent/discriminant validity testing it might be difficult to assess to what extent the Italian version of the BPQ measures body awareness and automatic reactivity” After this statement, I think the authors should address how this is particularly true for the BPQ Body Awareness subscale and there is a lot of debate regarding the different measurement models of interception. I know the authors introduce this issue subsequently, but this seems the ideal spot to introduce this issue.

**Response:** Thank you for your comment. We feel that, following the flow of the discourse, there is no need to change the order of the presented topics

Page 19

- “However, there is some evidence in contrast with the negative association we observed between physical activity and ANSR 37. Physical activity correlated negatively with the subscale Awareness, which is not consistent with other studies using different instruments of body awareness 37,38” – Two sentences addressing the same argument. Please revise.

**Response:** Thank you for your comment. Done.

- “In fact, Multidimensional Assessment of Interoceptive Awareness which is a questionnaire that measures interoception with a very different framework from the BPQ.” – This sentence is incomplete.

**Response:** Thank you for your comment. The sentence has been rephrased.

- “Interestingly being a smoker seems to correlate with a heightened awareness on the BPQ awareness subscale.” – Correlate may not be the most adequate term. Also written expression should be improved in this sentence.

**Response:** Thank you for your comment. Done.

- “ … the interpretation framework between the awareness of the interoception and smoking takes into account that the insula - which can be affected by nicotine making the interoceptive information available to conscious awareness.” – After reading it multiple times, I still cannot understand this sentence. Please revise it.

**Response:** Thanks for your comment. The manuscript has been revised by native English speakers.

**Page 20**

- “This result might be correlated with the findings from the study of Danner et al 46 in which the highest awareness …” – Again, correlated is not the most adequate term.

**Response:** Thank you for your comment. Done.

- “Our findings showed a positive correlation between ANSR Subdiaphragmatic subscale and total score and the use of psychoactive drugs. This finding is in line with previous studies 49” – Again, the authors stated in the reply to the revision that the total score would be removed, however they still debate it in the Discussion section. Furthermore, I do not understand how can the authors report a correlation with the use of psychoactive drugs, which is (if I understood correctly) a dichotomous variable. At the most the authors could compare drug users and non-users with an independent samples t-test.

**Response:** Thank you, done.

Sincerely,

The authors
