# Supplementary material for: Cross-cultural adaptation and psychometric properties of the Italian version of the Body Perception Questionnaire
Source: PLoS One. 2021 May 27;16(5):e0251838. doi: 10.1371/journal.pone.0251838 (PMC8158925; doi:10.1371/journal.pone.0251838)
Supplement: Attachment — Submitted filename: R3 Rebuttal Letter.docx [file pone.0251838.s005.docx]

**Review of the manuscript**

**Manuscript number PONE-D-19-35072, entitled “Cross-cultural adaptation and validity of the Italian version of the Body Perception Questionnaire.”**

Dear reviewers,

We greatly appreciate your detailed and thoughtful suggestions on the manuscript, that we acknowledge have improved the quality of the manuscript. A detailed description of all changes has been provided below.

For any further information, please do not hesitate to contact us.

**Reviewer 1**

Reviewer #1: The manuscript was fairly improved, as the authors completed most request minor changes. However, there are still major issues that should be addressed. Statistical reporting is still a major issue that needs to be improved so that the reader can clearly understand the psychometric properties of this questionnaire. Also, although the findings presented here partially support the BPQ-I as an effective tool to measure body awareness and autonomic reactivity, I believe that the current work has several limitations that are not clearly highlighted by the authors. This is extremely important to the field as there is a lot of debate about whether these measures are truly measuring what they are intended to measure. For instance, in the Abstract the authors argue: “Our results establish the BPQ-I for measurement of body awareness and experiences of autonomic reactivity” and this seems somewhat of an overstatement. Bellow, I provide my third round of revisions hoping that the authors can place their efforts to majorly improve the manuscript so that this relevant work can be published according to PLoS One standards.

Exploratory Factor Analysis: As suggested in my previous revisions, the authors added the exploratory factor analysis using the full-scoring system of the BPQ. This is extremely relevant when debating whether the BPQ is suitable to assess body awareness and autonomic reactivity. However, there are still several issues regarding this topic.

**Response:** Thank you for your work, we really appreciate your dedication and prior recommendations. The paper now has been revised to better match your requests and we do acknowledge that these have made for an improved manuscript.

1. The authors state “… we computed an EFA considering the same subscales and factors structure of CFA analysis”. This is quite confusing, as I do not properly understand the statistical procedures implemented for EFA analysis. Much more information should be provided to the reader. Also, using a priori factor structure for an exploratory analysis is not very advisable. The authors should clarify how did they implement the EFA.

**Response:**  While the initial plan for the analysis was to conduct a confirmatory factor analysis, we do agree that an exploratory factor analysis can provide information on alternate and optimal structure to the BPQ. We have now included comprehensive a detailed description of the EFA in the supplemental material, and these results are referenced in the main text.

2. The authors should also describe the importance of conducting an EFA for the full-scoring system in the Methods section, highlighting the problematic results reported by Cabrera et al. when they conducted a similar analysis.

**Response:**

3. The authors provided very limited results regarding the EFA and this is quite problematic. They should present to the reader which solutions were retrieved from these analyses, namely the number of factors and respective loadings. This would be critical to understanding whether the “untenably high numbers of factors and loadings with complex structure” found by Cabrera et al. was also an issue in this sample. Reporting these results clearly and transparently would be critical to researchers using these measures.

**Response:** We have now included a full description of the exploratory factor analysis conducted on the full-item distributions, which has now been included in the supplemental materials.

4. The EFA results for the full-scoring system should be ideally reported before the CFA for the binary scoring system. For instance, there would be no reason to use the binary scoring system if the EFA results provided a suitable solution for the full-scoring system, as the latter would provide much more sensitivity for individual differences.

**Response:** We agree with the reviewer that the EFA results may supersede the CFA. However, it is not advisable to conduct EFA and CFA on the same sample and our sample was not large enough to split into two sections. Based on our review of the literature and prior studies, as well as goals of conducting the CFA so that measurement invariance could be assessed (a goal that cannot be completed with EFA), the EFA must be considered a post-hoc analysis to test whether alternate factor structures are more plausible. Thus, the CFA is reported in the main section of the manuscript and the EFA in the supplemental material.

5. Confirmatory Factor Analysis: The authors did not make any adjustment regarding this previous comment: “The CFA procedures described in the Methods section are still quite incomplete. Further details should be given to the reader (e.g. type of matrix used, estimation procedure, are factors allowed to correlate or not, etc), at least in Supplement materials.”

**Response:** Thank you, we specified better all the features requested in the first paragraph of *Factor Analysis and Measurement Invariance Analysis (MIA)* (pag.10)and we added the factor loadings in S5 Table.

6. Regression Analysis: The authors completed several adjustments to the regression models. However, there are still a few issues that cannot be overlooked. For instance, why was physical activity only included as a predictor on one of the models? Also, more importantly, the authors still included predictors that are not suitable for analysis. There is still only 1 subject that reported Immunosuppressor drug use. This is not a valid variable to be included in a regression model and using stepwise regression models does not address this issue.

**Response:** Thank you for your suggestion. In the last manuscript we use a backward stepwise regression to select variables as predictors in our model. As you have well pointed out in some models physical activity was not included and other problems, as Immunosuppressant drug use, were still present. In order to improve our paper, we establish a-priori model that also includes the “physical activity” variable and we dichotomised the variable “use of drugs” in: medications use yes/no. We decided this strategy, as you suggest, considering the distribution of the responses.

7. Limitations, Recommendations for future studies & Convergent Validity: The authors added limitations and future studies heading, but the information provided there seems scarce. First, they should start that section by highlight the major limitation of the study: the lack of convergent and divergent validity. The results presented by the authors do not allow to affirm that BPQ-I is measuring body awareness and autonomic reactivity. This is particularly true regarding the body awareness subscale, as interoception-related measures have been widely questioned in the field. Thus, authors should clearly highlight this and recommend future studies to further explore this issue. Second, they describe that the current sample had high education levels, but they do not discuss the implications of this (e.g., is body-related phenom easily interpreted by participants with less education). Finally, the limitation regarding the EFA results was not clear to me, probably because the EFA implementation and results were not clearly described earlier.

**Response:** Thank you for your comment. We added an highlight of this issue in the limitation section as follows “*The present study includes some limitations. The psychometric evaluation of the questionnaire did not encompass the assessment of convergent and divergent validity. Therefore, it is not possible to affirm that the BPQ-I actually measures the intended constructs (i.e. body awareness and autonomic reactivity). Although the body awareness construct has been studied in previous studies [49], the autonomic reactivity construct still needs to be tested with sensor-based measures. Further studies are needed to better define those constructs and their properties. Participants were recruited from a pool of clients seeking osteopathic care. Therefore, the current sample is not a regular community sample, but rather an osteopathic care sample, where a significant percentage of subjects reported a health-related condition. It is possible that participants in this sample may have higher autonomic reactivity or lower awareness as part of their reason for seeking care. Further studies need to be conducted with more general samples to establish better normative data and replicate the psychometric features of the scale. Furthermore, the participants in the sample were highly educated (40% with a university degree). A previous study underlined how education level might impact the body awareness of subjects with a direct correlation [54]. However, the sample in the study was specific and therefore the results might be difficult to generalize. In order to understand the relationship between education level and body awareness further studies are needed.”*

8. Participants: In the first round of revisions, the authors argued: “… we have recruited subjects afferent to osteopathic care, and probably that may influence the selection of sample. If it is correct, we should expect a high presence of pathologies related to the osteopathic profession, which affect the musculoskeletal or neurological system.” In this second round of revision, the authors state: “ … Only 2% declared to have neurologic disorders, and we have no data concerning the musculoskeletal conditions of participants (which would fall eventually into “others disorders” that account for 5%). So we don’t feel we can support that statement.” This is quite contradicting. But mainly, the point of my revision regarding participant recruitment is that the current sample is not a regular community sample, but rather an osteopathic care sample, where a significant % of subjects report some sort of health-related condition as described in the Supplementary materials. This is a significant limitation of this study and should be clear for the reader in the Participants heading.

**Response:** Thank you for your comment. We added a sentence to specify that both in the “participants” paragraph and in the “limitations” paragraph. Respectively: “*Therefore, the current sample might not be considered a regular community sample, but rather an osteopathic care sample.*” and “*Therefore, the current sample is not a regular community sample, but rather an osteopathic care sample, where a significant percentage of subjects reported a health-related condition*”

9. Internal consistency interpretation: The values retrieved from Cabrera et al. should no be used to define criteria for internal consistency interpretation. Although not consensual, there are guidelines to interpret internal consistency values.

**Response:** Thank you for your comment. The mentioned section has been removed.

10. Internal consistency heading: The authors should make it clear that internal consistency was computed for each subscale separately, as requested in the previous revision.

**Response:** Thank you for your comment. We added the following sentence “*Internal consistency was computed for each subscale separately*”

11. Floor and ceiling effects: As presented in the previous revision, I do not believe that item 47 should be considered twice when assessing floor and ceiling effects.

**Response:** Thank you, we reconsider the items (46).

12. Incomplete information on sociodemographic and clinical variables: The author state “A specification of the timing of physical activity has been added to the table. 1. The “smoker” question did not ask for the number of cigarettes.” This should be clear not only in the Table but also when describing the online survey.

**Response:** Thank you for your comment. We included a better description of the questions in the proper online survey section: “Questions concerning medications use, physical activity and smoking habits allowed only dichotomous answers (yes/no) without quantitative specification, apart from the physical activity question (if ≥2/week)”

13. BPQ Description: “The BPQ description is still far too incomplete for a validation paper in my opinion. Although some information can be found in the Results section, I believe that this should be presented together with the scale description. The authors should describe how many items are included in each domain. Also, there is an item that is included in both the supradiaphragmatic reactivity and the subdiaphragmatic reactivity domains. This should be transparent for the reader. It should also be clear that separate scores are completed for the Body Awareness and Autonomic Reactivity domains (the BPQ manual does not even address a total score).” I believe that this comment, provided in the previous revision, was not still adequately addressed by the authors.

**Response:** Thank you for your comment! We created a specific section for BPQ description.

14. Cabrera et al. Citation: After reading the manuscript, I still feel that the Cabrera et al. citation is misused across the document. It seems that the rationale from some analytical methods was developed within this work which, sometimes, is clearly not the case. For instance, “based on the assumption that the instrument validly measures the construct to be measured1” (Page 8).

**Response:** Thank you for your comment. The assumption described in that sentence refers to the fact that Cabrera et al. studied the construct validity through factorial analysis. Indeed, the sentence specifically referred to the english and spanish version of the BPQ. Anyway, following your appropriate suggestion, all the references about Cabrera et al. were reviewed and where pertinent were changed accordingly.

15. Page 8 & 9: “The hypotheses were that the age was normally distributed with respect 9 to the Awareness subscale values and that age was negatively correlated with ANSR subscale value.” I still do not understand this hypothesis. Please clarify.

**Response:** Thank you for your comment. The hypotheses arose from previous published works. We added references for each hypothesis.

16. Page 8: “Consensus-based Standards for the selection of health Measurement Instruments (COSMIN) initiative 23 and the International Society for Quality of Life Research (ISOQOL) 22 have been used as conceptual framework”. I believe this information should be provided at the beginning of the heading.

**Response:** Thank you for your comment. Done.

17. Discussion about smoking: This paragraph needs to be revised. Although there were no differences between groups regarding the BPQ, the authors state that “the observed correlation converges with prior evidence and theory.”

**Response:**Thank you for your comment. The sentence has been changed as follow “*the observed correlation did not converges with prior evidence and theory*”

18. Statistical Reporting: There are still fairly unusual issues regarding statistical reporting For instance, using “p-value < 0.05” instead of simply using “p < 0.05”. Also, the authors should provide the exact p-value whenever possible.

**Response:** Thank you! Done!

19. Written expression: Written expression was widely improved across the whole document but there are still some issues that need to be revised. Bellow, there are some of these issues which I picked up.

**Response:** Thanks

20. Page 3

Original: “ … transmitted to the brain forming a neural pathway through …”

Suggested revision: “ … transmitted to the brain, forming a neural pathway through …”

**Response:** Thank you. Done.

21. Page 4

Original: “important sources of information that patient and clinician”

Suggested revision: “important sources of information that patients and clinicians”

**Response:** Thank you. Done.

22. Original: “trough efferent nerves that originate in the nucleus ambiguus in the brainstem”

Suggested revision: “trough efferent nerves that originate from the nucleus ambiguous in the brainstem”

**Response:** Thank you. Done.

23. Page 12

“Extensive information on the CFA is reported in Table 3.” Table 3 reports results from Measurement Invariance Analysis

**Response:** Thank you. Done.

24. Page 15

Original: “in accord to the factor structure described above”

Suggested revision: “in accordance to the factor structure described above”

**Response:** Thank you. Done.

25. Page 16

“Participants who were physically active were [HIGHER? LOWER?] than those who were inactive” - ???

**Response:** thank you, done!

Page 18

“Negative associations were found between ANSR subscale and age, physical activity, and

male gender, as well as and between age and male gender in the awareness subscale” – Rephrase this sentence

**Response:** Thank you for your comment. The sentence has been rephrased as follows “*Negative associations were found between ANSR subscale and age, physical activity, and male gender. Similarly it was observed a negative association between age and male gender and the awareness subscale.*”

We would like to sincerely thank the reviewer for the incredibly accurate revision of the manuscript that radically improved the overall quality of the work.

The authors
